# Increasing Venous Return Blood Flow to the Right Atrium Using the Party Balloon Inflation Maneuver

**DOI:** 10.1016/j.jaccas.2023.101997

**Published:** 2023-08-17

**Authors:** Akihisa Kataoka, Kento Kito, Kazuyo Shirakura, Taiga Katayama, Ken Kozuma

**Affiliations:** a Division of Cardiology, Department of Internal Medicine, Teikyo University, Tokyo, Japan; b Department of Clinical Laboratory, Teikyo University, Tokyo, Japan

**Keywords:** party balloon inflation maneuver, patent foramen ovale, Valsalva maneuver, venous return blood flow

## Abstract

The party balloon inflation maneuver increases intrathoracic pressure, decreases venous return, and after release enhances venous return to the right atrium more effectively than does the conventional Valsalva maneuver. Therefore, it shows potential for more effective detection of right-to-left shunts in patients with a patent foramen ovale. (**Level of Difficulty: Intermediate.**)

## Central Illustration

**Figure:**
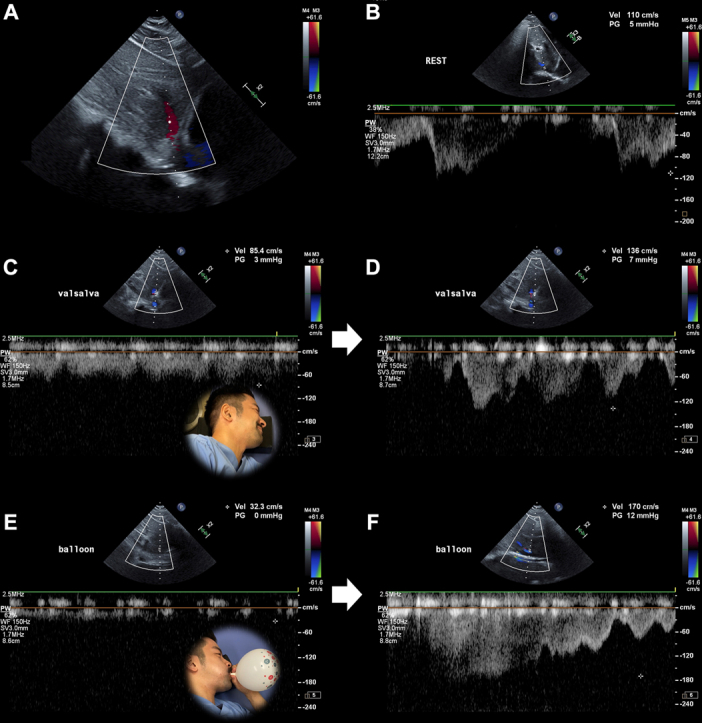

The Valsalva maneuver (VM) is widely used in the clinical setting because of its ability to induce hemodynamic changes.^1^ To diagnose a patent foramen ovale (PFO), VM is used in agitated saline contrast echocardiography to examine right-to-left cardiac shunting. VM release is characterized by an increased venous return to the right atrium (RA) and elevated RA pressure, resulting in enhanced right-to-left shunting through the PFO. However, a major issue with VM is the uncertainty regarding its effective application for both patients and examiners. False negatives have been reported in PFO detection cases wherein the VM is not properly executed.^2^

**Figure 1:**
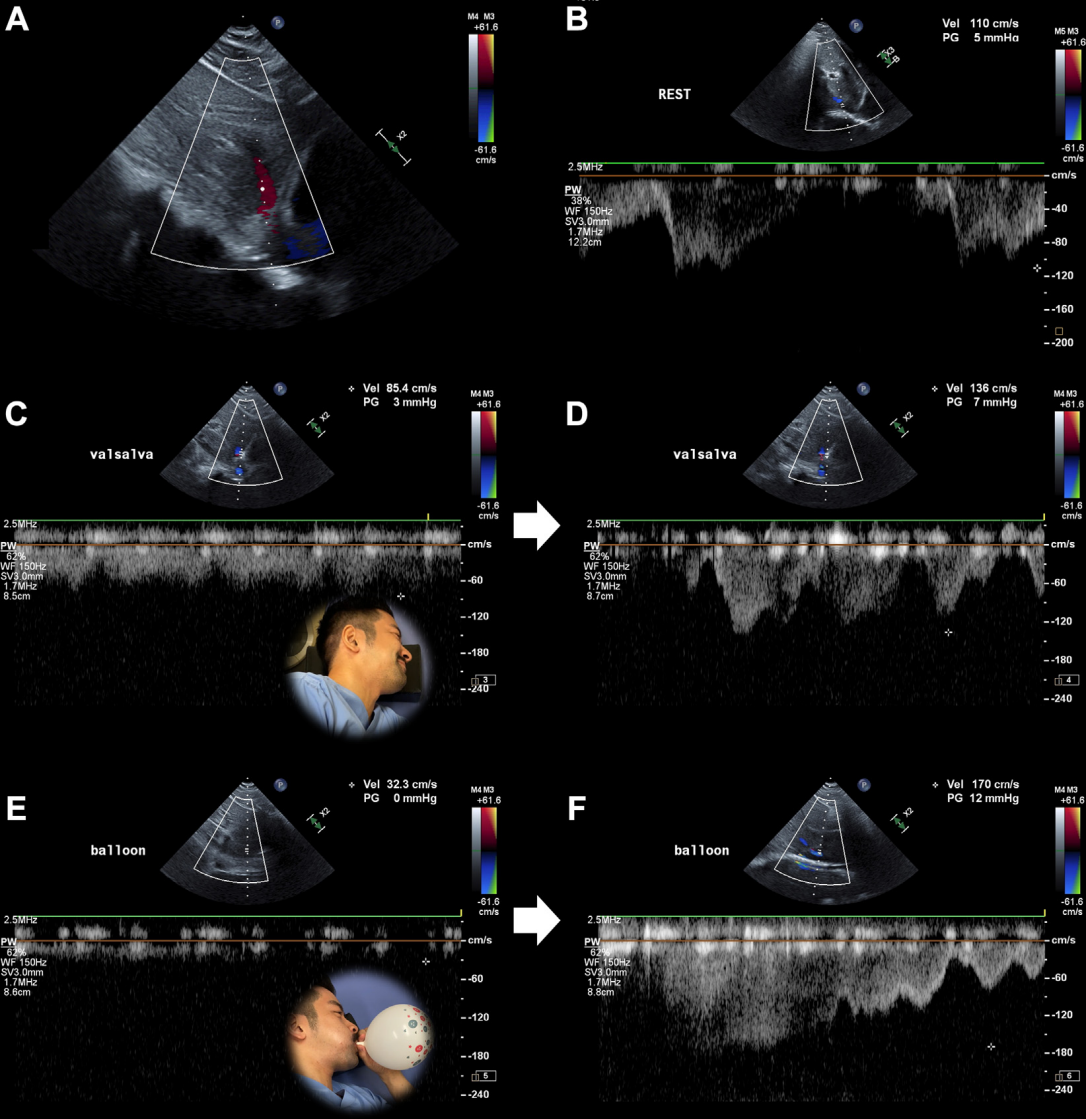
Color Doppler and Pulse-Wave Doppler of Hepatic Vein Flow **(A)** Color Doppler image of the hepatic vein. **(B)** Pulse-wave Doppler measurement of hepatic vein flow at rest shows a maximum flow velocity (MFV) of 110 cm/s toward the inferior vena cava. During the conventional Valsalva maneuver, **(C)** the MFV was reduced to 85.4 cm/s and **(D)** increased to 136 cm/s after release. With balloon inflation, **(E)** the MFV of the hepatic vein was further reduced to 32.3 cm/s and **(F)** increased to 170 cm/s after release (inspiration phase), indicating a substantial increase in hepatic vein flow compared with that of the conventional Valsalva maneuver.

Consequently, we developed the party balloon inflation maneuver (PBIM), which is useful for detecting PFO.^3^ The PBIM is easy to explain to patients and provides visual feedback to confirm the correct execution of the maneuver. However, the extent to which PBIM affects hemodynamics remains unclear. We conducted a demonstration comparing PBIM with conventional VM using the second author’s hepatic vein flow obtained through pulse-wave Doppler using the EPIQ CVx/S5-1t ultrasound system (Philips Medical Systems) (Figure 1A). Informed consent was not required because this was not a patient study.

During normal respiration (rest), hepatic vein pulse-wave Doppler showed hepatic vein flow toward the inferior vena cava, with a maximum flow velocity (MFV) of 110 cm/s (Figure 1B). After performing the VM, the positive pressure in the thoracic cavity caused the hepatic vein flow and MFV to reduce to 85.4 cm/s (Figure 1C). Upon release, the increased hepatic vein flow with 136 cm/s MFV indicated increased venous return to the RA (Figure 1D). Using the PBIM, the MFV was only 32.3 cm/s, and the hepatic vein flow was barely observable, suggesting a substantially stronger increase in intrathoracic pressure than that of the conventional VM (Figure 1E). Upon stopping inflation and initiating inspiration, a drastic increase in hepatic vein flow with an elevated MFV of 170.0 cm/s was observed, resulting in a substantial increase in venous return to the RA (Figure 1F).

The 30-mm-wide party balloon inflation provides 20 to 25 mm Hg of positive end-expiratory pressure to the patient’s airway,^3^ thereby increasing intrathoracic pressure, decreasing venous return, and enhancing venous return to the RA more effectively than does the conventional VM. Therefore, the PBIM approach may surpass the conventional VM in detecting right-to-left shunts in patients with a PFO.

## Funding Support and Author Disclosures

The authors have reported that they have no relationships relevant to the contents of this paper to disclose.
